# Survival of adult Steller sea lions in Alaska: senescence, annual variation and covariation with male reproductive success

**DOI:** 10.1098/rsos.170665

**Published:** 2018-01-17

**Authors:** Kelly K. Hastings, Lauri A. Jemison, Grey W. Pendleton

**Affiliations:** Alaska Department of Fish and Game, Division of Wildlife Conservation, PO Box 115526, Juneau, AK 99811, USA

**Keywords:** population dynamics, demography, vital rates, life history, mammal

## Abstract

Population dynamics of long-lived vertebrates depend critically on adult survival, yet factors affecting survival and covariation between survival and other vital rates in adults remain poorly examined for many taxonomic groups of long-lived mammals (e.g. actuarial senescence has been examined for only 9 of 34 extant pinniped species using longitudinal data). We used mark–recapture models and data from 2795 Steller sea lion (*Eumetopias jubatus*) pups individually marked at four of five rookeries in southeastern Alaska (SEAK) and resighted for 21 years to examine senescence, annual variability and covariation among life-history traits in this long-lived, sexually dimorphic pinniped. Sexes differed in age of onset (approx. 16–17 and approx. 8–9 years for females and males, respectively), but not rate (−0.047 and −0.046/year of age for females and males) of senescence. Survival of adult males from northern SEAK had greatest annual variability (approx. ±0.30 among years), whereas survival of adult females ranged approximately ±0.10 annually. Positive covariation between male survival and reproductive success was observed. Survival of territorial males was 0.20 higher than that of non-territorial males, resulting in the majority of males alive at oldest ages being territorial.

## 1. Background

Robust estimates of adult survival rates are necessary for modelling population change and fitness of vertebrates [1]. In long-lived birds and mammals, population trends are more sensitive to changes in adult than juvenile survival [2]. Incorporating variation in adult survival (e.g. due to age, environmental conditions, population density, individual quality) is important to predictive models [3,4]. For example, based on data from 58 mammalian species, ignoring senescence in adult survival in population viability models resulted in time to extinction overestimated by an average of 50% for species with high adult survival rates (more than 90%/year) [4]. Ignoring senescence in survival can also produce erroneous conclusions concerning demographic responses to density or environmental conditions [5].

Senescence is within-individual physiological deterioration with age, a principal cause of which is believed to be cumulative damage to biomolecules caused by oxidative stress [6]. Senescence has been detected in physiological variables, such as age-related changes in haematological measures [7] and reduced body mass with age [8]. Reduced health with age may be due to increased parasite burden [9], reduced immunity [10] and impaired cellular division with age (from damage to chromosomes' telomeres leading to cessation of cellular division, poorer tissue/organ functioning and reduced survival [11]). Reduced foraging efficiency may also occur with age [12], due to age-related decline in contractile ability of skeletal muscle [13] or damage to digestive system tissues from increased parasite burdens, which may reduce the ability to assimilate nutrients [14].

Actuarial senescence (decline in survival with increasing age, hereafter ‘senescence’) is common in wild populations of vertebrates [15,16]. Actuarial senescence is challenging to study in long-lived species, including marine mammals (examined in 9 of 34 extant pinniped species via longitudinal data), due to the need for large sample sizes to provide data of old ages, and for longitudinal data for unbiased studies [17]. Although female survival is particularly relevant for population dynamics of large mammals [18], male survival patterns are important to studies of life-history theory and fitness. In birds and mammals, adult survival is generally lower for the heterogametic sex (males in mammals, females in birds), probably due in part to expression of detrimental recessive alleles on their sex chromosomes [19]. Higher testosterone production may also cause greater mortality in males than females, because, unlike oestrogen, this hormone lacks antioxidant properties and has been linked to increased susceptibility to oxidative stress [20] and potentially, reduced immunocompetence [21] and survival [22].

In addition to intrinsic factors, sex differences in senescence are expected to be more pronounced in polygynous than monogamous species, with rate of male senescence expected to increase with sexual selection among males [23]. These expectations are based on two theories: (1) the sex suffering a greater rate of extrinsic condition-dependent mortality will have steeper senescent declines due to weakened selection against harmful mutations and antagonistically pleiotropic genes acting late in life [24,25]. The disposable soma theory (2) produces the same outcome, but predicts steeper declines in survival for the sex that experiences stronger intra-sexual reproductive competition due to somatic maintenance costs associated with competitive morphologies and behaviours related to the mating system [26]. Recent reviews present strong evidence for the disposable soma theory in particular and indicate sex differences in lifespan may largely result from sex-specific selection that optimizes life histories differently for males and females [16,25].

Steller sea lions (SSL, *Eumetopias jubatus*), occurring from California north around the Pacific Rim and into the Bering Sea, are polygynous, highly sexually dimorphic, and the most massive sea lion species [27]. From the late 1970s to 2000, SSL populations declined severely in the northern Gulf of Alaska and Russia [28,29] and were at only approximately 16% of historical numbers by 2004 in areas of greatest decline [30]. Geographically broad mark–resighting studies were initiated to provide survival estimates of SSL throughout their range, including precise age-specific estimates to ages 7–11 in Alaska [31–34] and to ages 15+ in Russia [35]. A sample of SSL marked during the severe population decline in Alaska and resighted to a maximum age of 16 could not be used to address age variation for adult years, because the high mortality resulted in very low sample sizes per age [31]. Data from SSL marked at the Kuril Islands, Russia during 1989 (*n*= 530) provide the only estimates of age-specific survival for older adult SSL (up to age 22; [35]). Rapid senescence in males greater than approximately age 11 and milder senescence in females after approximately ages 15–16 were observed, but precision of estimates at or above age 12 for males and at or above age 18 for females were poor, as fewer than 15 individuals remained at those ages (See electronic supplementary material, table S1 in [35]). In 1994–1995, 799 SSL were marked in southeastern Alaska (SEAK) and provide data to age 21 for comparison to Kuril Island estimates [35].

A faster rate and/or earlier onset of senescence in male than female SSL, as that observed by [35], are expected given levels of intra-sexual competition drive senescence patterns in polygynous species. However, effects of individual quality and territoriality on male survival are unknown. Age of onset of senescence of Russian males was similar to ages at which most SSL males hold breeding territories (ages 9–13 [36]). Successful males hold territories, often for more than 1 month, at rookeries for access to females in oestrus [37]. Maintaining territories is energetically costly: territorial males fast, are constantly vigilant, and may sustain injuries in fights with other males, potentially resulting in reduced survival of territorial versus non-territorial males. Therefore, negative covariation of survival and breeding success may be observed as earlier or greater breeding success associated with earlier onset or faster rate of senescence [15]. Although rarely observed, some adult males are killed in territorial conflicts [38]. Survival of non-territorial males that attempt to gain territories may also be compromised by aggressive interactions. Male survival may be positively correlated with reproductive success if the same phenotypic traits assist both processes (such as body size, behavioural repertoires, experience or foraging success [39–44]) and such positive covariation may produce high variation in fitness among individuals due to ‘individual quality’ [45].

Annual variability in adult survival may reflect changes in environmental conditions or population density. Such variability is expected to negatively affect the long-term population growth rate (reviewed by [46], but see [47]), and potentially create positive covariation among fitness components (i.e. poor conditions/high density lowers all fitness components although not necessarily by the same amount), which would probably magnify the effect of environmental variation on population change [48]. Understanding extrinsic drivers that produce survival patterns is useful both for determining best indicators of ecosystem change and also for building predictive models of population change [3]. Although adult female survival is expected to be less temporally variable than juvenile survival or reproductive rate of young females [2], adult survival may respond strongly to environmental variability [49] and density dependence [50]. Particularly, oldest ages [2] and males [51] may exhibit high annual variation in survival or be more affected by density dependence. Understanding annual variability in survival of adult SSL is needed for population models, to assess extrinsic drivers of population change, and to determine best indicators of ecosystem change. Our objectives were to: (1) estimate age- and sex-specific survival of SSL to age 21, (2) examine annual variation in survival of adult male and female SSL in northern and southern SEAK, and (3) determine if territorial state (and assumed reproductive success) was positively or negatively associated with survival in adult male SSL.

## 2. Material and methods

**Figure 1.**
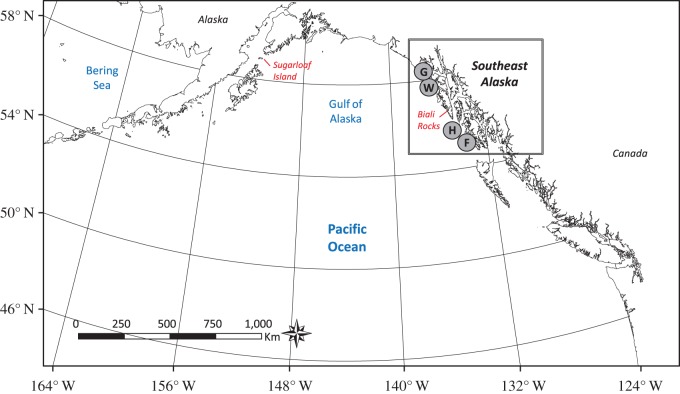
Map of SSL rookeries in SEAK. F,Forrester Island complex; H,Hazy Islands; W, White Sisters; G, Graves Rocks. Biali Rocks and Sugarloaf Island are also shown.

From 1994 to 1995 and 2001 to 2005 the Alaska Department of Fish and Game (ADFG) branded 2795 SSL pups on their left sides with unique alpha-numeric sequences in late June–early July (2–4 weeks of age) at four of five rookeries in SEAK [31,32]. Pups were marked at Forrester Island complex (consisting of 5 islands at which females pup) in 6 years (F, 141–400/year, total = 1,795), Hazy Islands in 3 years (H, 101–225/year, total = 539), White Sisters in 3 years (W, 94–147/year, total = 368), and Graves Rocks in 2 years (G, 43–50/year, total = 93; figure 1). Animal capture, branding and sampling procedures were approved by permits for marine mammal research activities issued to ADFG by the US National Marine Fisheries Service.

Branded SSL were observed and photographed each year from 2000 to 2015 during 1–3 dedicated boat-based surveys per summer (May–July), which were standardized in that all rookeries and major haul-outs in SEAK were visited at least once [31,32,52]. The order and the date sites were visited varied at most approximately 2 weeks among years. Northern British Columbia, Canada was surveyed via dedicated surveys in 11 years and by opportunistic sightings. Miscellaneous photographs of branded animals were also provided by other entities (especially the Alaska Fisheries Science Center (National Marine Fisheries Service), Alaska SeaLife Center, Glacier Bay National Park and the North Pacific Universities Marine Mammal Research Consortium), extending geographical-coverage from Oregon to the Bering Sea and Russia.

To address the assumption of mark–recapture models that marks are not lost or misread, we used only resighting data that were accompanied by photographs from which identities of animals were confirmed against a master photograph library maintained by ADFG [32]. By rigorous review process and inclusion of only photograph-confirmed resightings, assignments to individuals were consistent over time for both clearly legible and misshapen brands, and all clearly legible brands were accurately assigned to individuals. All occurrences of misshapen brands were included, given photographs were of good quality and provided good views of brands. Therefore, mark loss did not occur but misshapen brands required careful treatment. Misshapen brands were either assigned to the correct individual based on additional information (birth year, birth location and sex + process of elimination (other animals that were possible matches were also observed), *n* = 100 of 1207 individuals seen after the birth year, 8.3%) or were randomly assigned to individuals based on available information (for those few cases in which process of elimination could not resolve identification to individual, *n *= 39 of 1207, 3.2%). For these 39 randomly assigned, most (30 of 39) were seen as adults and sex was, therefore, known, most were from the 1994–1995 cohorts (30 of 39), and birth location and year were known for all but five who could not be identified as born in either 1994 or 1995. Of the 100 with misshapen brands that could be resolved to individual, 58 were females and 42 were males suggesting a slightly higher rate of misshapen digits for females than males or similar rates for both sexes. Given only 3.2% of data required this assignment and of these most were of certain sex, we expect effect of this reassignment on estimates was negligible and allowed brand loss to be properly accounted for to prevent bias in survival estimates.

Resighting data of the 1994–1995 cohorts collected before 2000 were geographically limited and not documented with photographs [31], and, therefore, were not used. Resighting effort after 2000 was especially high at F because data from an annual field camp from May to July each year provided near-daily surveys, (except in 2006, when court injunction prevented SSL field research in the USA from May to June). Behaviours (e.g. territoriality in males) of branded animals were recorded when observed. Because at rookeries during the breeding season non-breeding males are segregated from females, males present among one or more females for extended periods (usually 1+ hour) were deemed territorial. Very often mating and/or territorial defence behaviours such as display behaviour and fights with neighbouring territorial males or unsuccessful male intruders were also observed.

Survival estimates for ages 13+ were from two cohorts (1994–1995). For these cohorts, estimates were possible only from ages 5 to 21 because collection of resighting data started in 2000, and therefore only cumulative survival from birth to age 5–6 could be estimated. The 2001–2005 cohorts provided estimates from birth to ages 9–13. Eleven individuals (less than 0.5% of animals) were randomly assigned a sex for data analysis because sex was not recorded at marking and resighting data also did not provide sex information because the animals had not survived to older ages when sexual dimorphism allows unequivocal sex determination.

For mark–recapture modelling, we used Cormack–Jolly–Seber (CJS) [53] or multi-state models (MS) [54] and program MARK [55] with the RMark interface [56] to fit models to data and estimate parameters (CJS/MS = probabilities of survival, *S*, and resighting, *p*; MS only = probabilities of changing states, *ψ*). Before fitting models, we assessed goodness-of-fit of the most complex model using the median $\hat{c}$ procedure in MARK, and used the estimated $\hat{c}$ to adjust results if significant overdispersion $\left(\hat{c}\;\gg 1.0\right)$ was indicated [57]. We did this to address the assumption of the CJS model that *S* and *p* were sufficiently homogeneous among individuals. Best models were determined by model weight based on Akaike's Information Criterion corrected for small sample size (AICc or QAICc with overdispersion correction) [57].

### 2.1. Senescence in survival

We created capture histories of 18 occasions (1994, 1995, 2000–2015) for all branded individuals by treating multiple sightings of an individual per summer (June–August at haul-outs or May–August at rookeries) as a single annual observation. We used resighting data collected over a 3–4 month timespan and estimated *S* over 12 month timespans (annual *S*) to address the assumption of the CJS model of ‘instantaneous’ sampling relative to the survival interval being examined.

Using the CJS model, we fit three candidate *S* models separately using the same 171 *p* models. The base of the *S* model was the best *S* model from [32]: age × sex + natal rookery (nr) + cohort_py (cohort effect fit only to pups and yearlings, and the same for pups and yearlings). This base model was used because the same resighting data for the juvenile and early adult years (to age 9) and animals were used as in [31] and [32]. We fit more models based on this initial model, to address variation during the later adult years not previously possible. We also included in all *S* models two cumulative periods mentioned previously (birth to age 6 for the 1994 cohort and birth to age 5 for the 1995 cohort) for males and females separately (four separate parameters) to account for the period without photograph-based resighting data (before 2000). *S* model 1 included age as all separate annual estimates (age 0–21). Model 2 included age as seven categories with female categories (ages 0, 1, 2, 3–14 [constant annual *S* for ages 3–14], 15–16, 17–18, 19+) differing from those of males (0, 1, 2, 3–7, 8–11, 12–14, 15+). We fit model 2 based on results of [32,33] which found *S* differences for ages 0–3 but then a period of constant *S* at age 3+ for both sexes. We pooled later ages *a priori* in model 2 to assist smaller sample sizes at later ages, earlier for males than females based on results from [35]. Model 3 included age (differently for males and females) using a B-spline (basis spline) matrix with four degrees of freedom, which used piecewise polynomials to provide a complex nonlinear pattern with age using fewer parameters than the other two models [35].

The basis of *p* models was the best *p* model from [32]: age × sex + year, with all ages estimated separately (model 1). Because that study only used data to age 7/8, we also fit models including age categories to span 22 years (models 2–9 for all possible combinations of two additional structures for females and four additional structures for males). We fit two additional structures for females based on estimates in [32] which found *p* differed from ages 1–3 but was similar at ages 4–8, and suspected potential reduced *p* at older ages when *S* may also decline [35] (*a*: 1–3 separate, 4–14, 15+; *b*: 1–3 separate, 4+). We fit four additional structures for males which showed a more complex age pattern to age 8 than females (possibly 1–8 all different or 5–8 similar [32]) and also included models allowing lower *p* for older males but at earlier ages than for females [35] (*a*: 1–8 separate, 9–12, 13+; *b*: 1–8 separate, 9+; *c*: 1–5 separate, 6–12, 13+; *d*: 1–5 separate, 6+). In addition to age categories, we also reconsidered effects of nr and nr × year on *p* given later adult ages were now available. Potential models with nr effects included: (1) additive nr effect the same for all ages; (2) additive nr effect only for adult females; (3) additive nr effect for adults both sexes; (4) nr × year effect the same for all ages; (5) nr × year effect differing from juveniles and adults; and (6) nr × year effect only for adult females. For models including nr effects, we considered three categories for nr: (1) all separate F, H, W and G; (2) F, H, WG pooled (due to small sample sizes at G and similar dynamics at W/G [32]); and (3) F, HWG pooled (due to particularly high effort at an annual field camp at F). All combinations of models based on these nr effects, nr categories and age structures (no nr effect = 9 models + nr effects = 6 × 3 × 9 models) resulted in 171 *p* models considered.

### 2.2. Annual variation in adult survival

Annual variation in *S* of adults (defined as ages 3+ by constant high *S* after that age, until senescent effects [32,33,35]) was then added to the best model from §2.1 as 16 additional models. We considered four models for females aged 3+ (*a:* south only [nr = H and F], *b*: north only [nr = W and G], *c*: differing north/south, *d*: same north/south), eight models for males (four each for males aged 3+ and 9+: same as for females), and four for animals aged 3+ sexes pooled (same as for females). We pooled sites into north (WG) and south (FH) because dynamics differ between north and south and are similar within north and south [32]. For example, *S* of juveniles was higher for animals born in the north than in the south, and nearly identical for animals born at F and H [32]. Pooling of WG was also required due to small sample sizes at G. We considered males aged 9+ specifically when sharp declines in *S* of males may occur [35].

### 2.3. Territoriality and correlation with male survival

Annual capture histories for males pooled multiple sightings from May to September in a year into a single sighting by best location × territorial ‘state’ in that year. Instead of coding 1 (seen) and 0 (not seen) used for CJS models, for MS models, ‘1’ was replaced by states A (held prime-season territory at F, beginning before 19 June), B (late season at F, beginning after 18 June and before 1 July), C (very-late season at F, beginning after 30 June, see electronic supplementary material, Report S1: Breeding behaviour of males), N (non-territorial at F), T (at least one sighting territorial other rookery) or H (only seen non-territorial at other rookery or at haul-out). Best state for that year was determined as N > H; all other codes were exclusive. The informative comparison was states A/B/C versus N, with a control for T/H, because territorial behaviour recorded at rookeries other than F was not useful due to only brief surveys after 21 June at other rookeries and haul-outs (see electronic supplementary material, Report S1). Males from the 1994–1995 cohorts were of breeding age before 2007, but were considered of unknown state before 2007 due to insufficient survey effort. A few males from the 1994–1995 cohorts remained by 2007; the 2001–2005 cohorts contributed the most data.

The basis for *S* and *p* models was the best model for males from §2.1, with additive effect of state also in *p*. A single *p* model was fit because we expected *p* varied by state due to high survey effort at F and to higher *p* of territorial versus non-territorial males. We first modelled *ψ* using a base model state × to state × nr3 × age, where nr3 was natal rookeries: F, H and pooled WG (due to small sample sizes). Age was included as: ‘age3’ (5–7, 8–9, 10+ years), ‘age2’ (5–9, 10+) or no age effect. *ψ* was fixed to 0 for ages 0–4 as males were not territorial at these ages. The best *ψ* model was then fit to 7 *S* models which pooled states in *S*. Because fitting effects for A, B and C separately was not possible due to small sample sizes of these states, modelling was done separately for three datasets in which ABC was pooled various ways with N. Finally, we considered $\hat{S}$ and $\hat{\psi }\;$ from the best model together to estimate age-specific proportions of F-born males that were territorial at F.

## 3. Results

### 3.1. Senescence in survival

**Figure 2.**
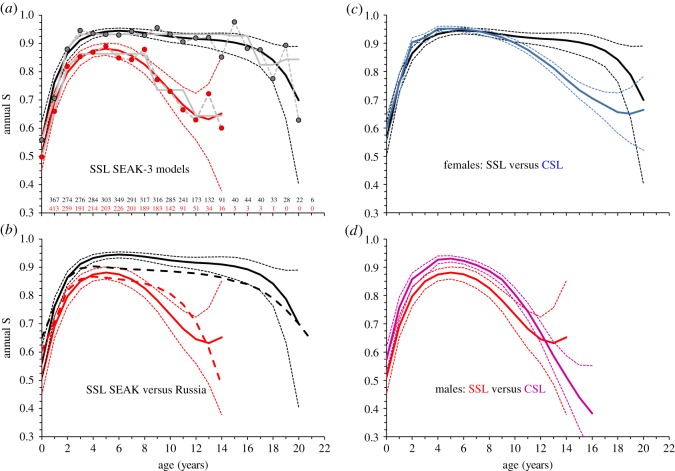
Estimates of age-specific survival probabilities (S) for female and male SSL born at Forrester Island complex (F), SEAK to 21 years of age using data 2000–2015, with comparisons to other studies. Number of animals resighted per age: see bottom of (*a*) for males (red) and females (black). (*a*) Estimates for SSL from F from the three survival models plotted together: dotted grey line with filled circles: estimates from all ages separate model (model 1); bolded grey lines: from age categorical model (model 2); bolded coloured lines (coloured dotted lines = 95% CI): from B-splines model (model 3). Males (red), females (black). (*b*) Estimates for SSL from F (bolded coloured lines with 95% CI) versus Kuril Islands, Russia (dashed lines, estimates from [35]). Males (red), females (black). (*c*) Estimates for female SSL from F (black) versus female California sea lions (CSL) from San Miguel Island, California (blue); 95% CI are shown for both, CSL estimates from [58] for ages in which *n* ≥ 30. (*d*) Estimates for male SSL from F (red) versus male CSL from San Miguel Island, California (pink); 95% CI are shown for both, CSL estimates from [58] for ages in which *n* ≥ 30.

Less than 6 males remained after age 14, compared to 44–22 females per age from ages 14–20 (figure 2*a*); thus age-specific *S* for males could not be estimated after age 14 (confidence intervals ranged from 0 to 1), and we fit a B-spline only to ages 0–14 for males with *S* at age 15+ pooled as a separate parameter. A $\hat{c}$ of 1.25 indicated mild lack of fit for the most complex model and was used to adjust results.

The best *p* model was age × sex + nr (adults only) + year (electronic supplementary material, table S2-A). Age as categories was preferred, but because no single best age structure was apparent (QAICc weight of top five models ranged 0.06–0.11), we used the most complex structure occurring in the top five models (model 32: electronic supplementary material, table S2-A) for modelling *S.* Categorical age was also strongly supported for *S* (QAICc 754–756 for all separate *S* versus 722–724 for ages categorical), but the *S* model with age fit using B-splines with a few less parameters was most preferred (QAICc 720–722 for B-splines model, electronic supplementary material, table S2-A). $\hat{S}\;$ (B-splines model) closely fit those from the full age × sex model, and had high precision for all ages but the oldest ages (figure 2*a*, electronic supplementary material, table S3). Senescence in *S* for F females was most apparent after age 17 with $\hat{S}$ from the categorical model indicating a 0.11 drop in $\hat{S}$ from ages 3–15 to 17–19 (figure 2*a*). $\hat{S}$ of F males was lower than that of F females, as expected, and with earlier senescence, particularly after age 8 (figure 2*a*). Based on the B-splines model, male $\hat{S}$ ranged 0.85–0.88 at ages 3–7 and then dropped an average of 0.05/age from ages 8–12 to a low of 0.67 for ages 12–14 (figure 2*a*). Using the *S* model with categorical age (model 2), senescence in *S* for both sexes was strongly supported (QAICc weights: 0.76, 0.23 and 0.01, respectively, for models with senescence for both sexes, only in males, and only in females; electronic supplementary material, table S2-B).

### 3.2. Annual variation in adult survival

**Figure 3.**
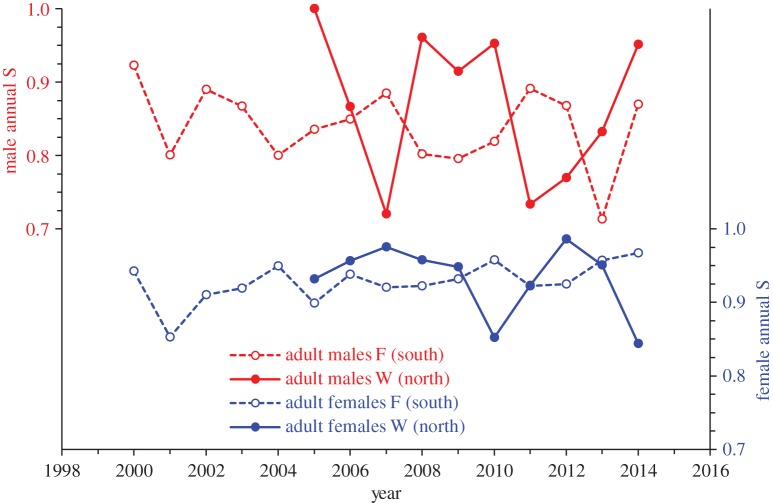
Annual variation in survival probabilities for female (blue) and male (red) SSL born in southern (Forrester Island complex, F) and northern (White Sisters, W) SEAK. Estimates from models 174 and 178 in electronic supplementary material, table S2-C. Estimates shown are at age 3 (survival was constant and high at ages 3+ for both sexes, until senescent effects [32,33,35], figure 2).

Overall annual variation in *S* was statistically supported only for northern-born males aged 3+ (QAICc weight 0.79 versus 0.10 next best model, electronic supplementary material, table S2-C). $\hat{S}$ suggested higher annual variation in males than females, and in the north than south for both sexes, and that the annual pattern may have differed between sexes (electronic supplementary material, table S2-C, figure 3). Annual $\hat{S}$ ranged ±0.07 and ±0.14 for females born at F and W, versus ±0.18 and ±0.28 for males born at F and W (figure 3).

### 3.3. Territoriality and correlation with male survival

Seventy-one males were territorial at rookeries other than F from 2000–2015, including two at Biali Rocks, 22 at H, 18 at G, 28 at W and 1 at Sugarloaf Island (figure 1). All held territories at only one rookery. Fifty-three branded males were territorial at F from 2000 to 2015 (electronic supplementary material, Report S1), and none were observed territorial at different islands at F or at other rookeries. Most males held territories at their natal sites (0.60–0.78 except 1.00 for G-born males, table 1). W-born males held territories only at G and W, and H-born males had the least natal fidelity and were most dispersed (table 1). $\hat{S}\;$ were much higher for territorial than non-territorial males at F, particularly for males holding late or very-late season territories (table 2, electronic supplementary material, table S4). $\hat{S}\;$ of territorial versus non-territorial males at F was +0.20, +0.12 and +0.05, respectively, for ABC/N, AB/CN and A/BCN, at age 10 (table 2). $\hat{S}\;$ was similar between the two groups at other rookeries and to non-territorial males at F (N) such that pooling of N/H/T was appropriate (table 2, electronic supplementary material, table S4). By combining $\hat{\psi }$ and $\hat{S}$ from model 6 (electronic supplementary material, table S4) for datasets 1 and 3 ($\hat{\psi }$ in electronic supplementary material, table S5), almost no F-born males had territories before age 9 at F, and the majority of F-born males alive at older ages (12+), were territorial at F. Estimated proportions of F-born males alive at each age that held prime-season territories at F at that age were 6% (age 9), 12% (age 10), 39% (age 11), 51% (age 12), 58% (age 13) and 63% (age 14). Age structure shifted younger when late-season territories were included: 1% (age 7), 2% (age 8), 22% (age 9), 36% (age 10), 57% (age 11), 61% (age 12), 66% (age 13) and 71% (age 14).

**Table 1.** Breeding rookery fidelity of adult male SSL from SEAK by natal rookery. For males from each natal rookery (NR), proportions are numbers of males observed at territorial rookeries (TR)/the total number of males ever observed territorial. B, Biali Rocks, F, Forrester Island complex, H, Hazy Islands, G, Graves Rocks, W, White Sisters, X, Sugarloaf Island (figure 1 for locations). Proportions of males territorial at their natal site are bolded.

	TR						
NR	B	F	H	G	W	X	*n*
F	—	**0.78**	0.11	—	0.11	—	62
H	0.11	0.06	**0.60**	0.11	0.06	0.06	18
G	—	—	—	**1.00**	0	—	10
W	—	—	—	0.25	**0.75**	—	24

**Table 2.** Estimates of survival *(95% CI)* from age 10 to age 11 for SSL males born at Forrester Island complex, SEAK, based on territorial status. T(F), territorial at F; A/B/C, prime-/late-/very-late-season territories at F. N, non-territorial at F. ‘T(F) = All’: A/B/C versus N. ‘T(F) = prime + late’: A/B versus N/C. ‘T(F) = prime only’: A versus N/B/C. Only males holding late- or very-late-season territories were available at rookeries other than F (T = late + very late only).

	T(F) = All	T(F) = prime + late	T(F) = prime only
territorial at Forrester, T(F)	0.821 (*0.664–0.914)*	0.791 *(0.613–0.900)*	0.748 *(0.541–0.883)*
non-territorial at Forrester (N)	0.625 (*0.470–0.758)*	0.671 *(0.522–0.791)*	0.703 *(0.562–0.814)*

	T = late + very late only		
territorial at other rookery (T)	0.606 (*0.470–0.727)*		
non-territorial, other rookery/haul-out	0.665 (*0.577–0.743)*		

## 4. Discussion

Senescence in *S* of females from SEAK had strong statistical support. Although $\hat{S}$ of adult F-born females were greater than those of adult females from the Kuril Islands, onset and rate of senescence were similar for both studies. Senescence was apparent at ages approximately 16–17 and rates of change per age after age 16 averaged −0.047 and −0.040 (absolute value, calculated from spline-based estimates in figure 2*b*) in SEAK and Russia, respectively [35]. Assuming rate of senescence observed for this study continued over a lifespan of 30 years, a simple Leslie matrix model suggested ignoring actuarial senescence of females would slightly overestimate population trend ($\hat{r}$). We fit a population model using $\hat{S}$ from this study (but substituting first-year *S* from [59] to include mortality from birth to 3 weeks of age and an average $\hat{S}$ from ages 3–16) and reproductive rate of 0.35 female pups produced per adult female per year for females aged 5–30 (see [59]). This model produced an $\hat{r}$ of +0.011 without senescence in $\hat{S}$ (using average $\hat{S}$ from ages 3–16 to age 30), and −0.003 by the model with senescence at the rate observed in this study at age 16+.

Onset of male senescence was earlier in our study (ages 8–9) than in Russia (approx. ages 10–11) (figure 2*b*). It is possible rate of male senescence was higher in Russia than in SEAK with average rates of decline in *S* per age of −0.080 (after age 11) and −0.046 (after age 8), respectively (from spline-based estimates, figure 2*b*). Small sample sizes of older ages as well as few cohorts marked per area (two in SEAK and one in Russia) prevent a true comparison of area-specific patterns. Owing to low overall survival and earlier onset of senescence, but perhaps not a greater rate of senescence, the lifespan of male SSL is particularly short. Similarly, in wild boar (*Sus scrofa*), male mortality rates overall were higher than those of females, but rates of senescence were similar for both sexes [60]. Both age of onset and rate need to be considered in senescence studies [61]. Delayed onset of senescence in females relative to males may be due to condition-dependent mortality affecting males throughout their lives but affecting females only at older ages. For example, in male Soay sheep (*Ovis aries*), parasite burden increased linearly with age in males but non-linearly in females, affecting only oldest females [9]. Many studies demonstrate a steeper rate of senescence in males than females [62]. Because only a few cohorts were marked in SEAK and Russia, differences in senescence patterns of males between areas may reflect early conditions (i.e. cohort effects) which can affect senescence patterns [63].

Patterns of senescence observed in other pinniped species to date are diverse, but studies are few and many have limited sample sizes of older ages (nine species with longitudinal data). Age of onset of senescence in female SSL was similar to northern elephant seals (*Mirounga angustirostris*, ages 16–21 but sample sizes were less than 15 individuals after age 8 [64]), Hawaiian monk seals (*Monachus shauinslandi,* ages 17–18 [65]), and New Zealand sea lions (*Phocarctos hookeri*, $\hat{S}$ = 0.90–0.95 from ages 10–15 and 0.80 by age 20 [66]). Onset of senescence in females was earlier (age 13) in subantarctic fur seals (*Arctocephalus tropicalis*) than SSL [45]. Also in that species, rate of senescence was much higher for non-breeders than breeders, producing positive covariation in survival and breeding success in females [45]. Senescence in female California sea lions (*Zalophus californianus*) born in Mexico was detected at ages 10+ ($\hat{S}$ = 0.91, down from 0.97 at ages 5–9), but specific age of onset could not be determined [67]. A uniquely large and long-term dataset was available for California sea lions born at San Miguel Island, California where over 11 000 pups were marked and resighted over 28 years [58]. Senescence in female California sea lions was earlier than in female SSL, with survival declining after age 11, and the rate of senescence was potentially lower in California sea lions compared to SSL [58] (figure 2*c*). Actuarial senescence was not detected in females to age 20 in Antarctic fur seals (*Arctocephalus gazella*, but sample sizes were less than 25 after age 14) [68], nor in southern elephant seals (*Mirounga leonina*) [69,70], and Weddell seals (*Leptonychotes weddellii*) [71,72] despite large, long-term datasets.

Survival patterns of male SSL were most similar to those of California sea lions (also highly sexually dimorphic and polygynous). $\hat{S}$ of male California sea lions born at San Miguel Island, California peaked at age 5 (0.93) and dropped below 0.90 by age 8 and below 0.70 by age 12 [58] (figure 2*d*). Similarly, for this species in Mexico, $\hat{S}$ declined from 0.90 at ages 5–9 to 0.75 at ages 10+, but age of onset could not be resolved [67]. Age of onset of senescence (ages 17–18) was much later in male Hawaiian monk seals than in male SSL, and was the same for both sexes in monk seals [65]. Senescence was not apparent in male northern elephant seals from ages 1–15, but average $\hat{S}$ of males was exceptionally low (0.66–0.72), and sample sizes were less than 15 after age 5 [64]. Senescence was also not observed in male Weddell seals to age 17 despite a large, robust sample [71]. Both Hawaiian monk seals and Weddell seals are only moderately polygynous and sexually dimorphic. Therefore, males in highly polygynous and markedly sexually dimorphic species, with sufficiently high survival rates to allow longevity, may experience a high rate or early onset of senescence as predicted from life-history theory.

We may expect both lower and greater annual variability in survival in the most vulnerable or energetically challenged demographic groups [2]. Based on spatial patterns in SEAK, lower overall survival was not associated with greater annual variability in survival. We expected greater annual variability in southern-born than northern-born SSL, because lower survival of southern-born SSL may have indicated a less productive and/or less predictable environment in the south or that higher animal density reduced prey availability [32]. High population growth in the north and population stability in the south [73] also suggested a potentially more productive or safer environment in the north. The newly established rookeries in the north are a unique zone of stock-mixing, and result from recent colonization by breeding SSL from both Alaskan stocks [74]. However, overall annual variation in survival was greater in northern than southern SEAK in both sexes, with statistical support only for males (figure 3). Greater variability in fitness components of SSL in the north may be due to a greater reliance on temporally or spatially variable but also potentially more abundant or nutritious prey. More studies are required to determine the role of prey conditions versus other mortality factors (disease, toxins, predators, detrimental human interactions) in shaping the annual and spatial patterns observed.

Greatest annual variability in adult survival was observed in males aged 3+ from northern SEAK (ranging nearly ± 0.30 among years), suggesting this demographic group may be the most sensitive indicator of environmental conditions, if this pattern is due to prey availability rather than other causes. Male survival, in general, may be more condition-dependent than female survival [51]. This may be particularly true in SSL in which body mass of adult males is 2.5× larger than adult females due to rapid and prolonged body growth in males until age 8–9, which is also coupled with high seasonality for growth in males (occurring mainly November–February) especially above age 5 [75]. Therefore, survival of males is probably dependent on winter forage conditions. Greater variability in males than females may also be due to more extensive movements of males [52], which may expose them to a diversity of conditions and hazards. However, movements of males born in the north were reduced compared to those for males born in the south (LA Jemsion 2017, unpublished data), suggesting movement propensity cannot be responsible for the spatial variation in the annual survival patterns of males. Subadult and adult male California sea lions were especially susceptible to disease conditions (possibly due to sex differences in geographic distribution) which may have caused or contributed to annual variation in male survival [58].

Unlike Hawaiian monk seals in which temporal variation in adult survival was similar among sexes [65], none of the four groups in our study showed similar annual patterns, suggesting extrinsic drivers may differ between sexes and geographical areas (figure 3). Annual survival of adult females ranged within approximately ±0.10 among years (figure 3), similar to Hawaiian monk seals (both sexes, approximately ±0.10–0.20 at ages 5+, figure 5 in [65]) and New Zealand sea lions (females aged 4–15, ±0.06 [76]). In contrast, high annual variation in survival (ranging nearly ± 0.30 among years) was reported for adult female Antarctic fur seals (which also had low average survival at 0.83), and annual survival probabilities were weakly correlated to annual pup growth rates, suggesting a food link [68]. The lower annual variability in adult female survival probabilities we observed for SSL suggests female SSL may use a risk-avoiding strategy (e.g. flexible reproductive output to conserve their own survival) to buffer environmental variability [46]. However, the underlying drivers of the annual patterns we observed remain unknown and require further study.

No territorial males established territories at more than one rookery during our study, suggesting breeding rookery philopatry was approximately 1.0. In contrast, natal philopatry ranged 0.60–1.0 (table 1), but observations were late-season at H, W and G, and so may represent ephemeral behaviour by males that never held prime-season territories. In comparison, SSL females from SEAK had breeding philopatry of 0.973–0.996 and natal philopatry of 0.776–1.00, ranging among the four major rookeries in SEAK [77].

We observed positive covariation between survival and territoriality in males (table 2). This covariation resulted in a majority of F-born males territorial at older ages, such that at age 14, 63% held prime-season territories and 71% held prime- or late-season territories. Similar positive covariation has also been observed in male fallow deer *Dama dama*, bighorn ewes *Ovis canadensis*, and possibly older male northern elephant seals [40,42,78]. Although territoriality does not ensure reproductive success [37], the likely positive covariation between survival and reproductive success in SSL males suggests that a greater proportion of phenotypically above-average males at older ages may provide strong selection for male behaviours and traits and possibly the best mates for adult females [42].

## Supplementary Material

Supplemental Report S1: Breeding behavior of male Steller sea lions in southeastern Alaska, 2000 – 2015

## Supplementary Material

Supplemental Table S2. Model selection results for estimating age-specific survival of Steller sea lions in southeastern Alaska to 21 yrs of age (A), with assessment of statistical evidence of senescence (B) and annual variation (C) in adult survival, using the Cormack-Jolly-Seber model.

## Supplementary Material

Supplemental Table S3. Age- and sex-specific survival probabilities (S ^) of Steller sea lions in southeastern Alaska by natal rookery.

## Supplementary Material

Supplemental Table S4. Model selection results for survival differences between territorial and non-territorial Steller sea lion males in southeastern Alaska (2007–15) using the multi-state model.

## Supplementary Material

Supplemental Table S5. Estimates of the probability of Steller sea lion males transitioning between territorial states (ψ) in southeastern Alaska, 2007–2015 by age and natal rookery.

## Data accessibility

Data from this study are available on Dryad. (http://dx.doi.org/10.5061/dryad.541r5) [79].
