# Supplementary material for: Survival of adult Steller sea lions in Alaska: senescence, annual variation and covariation with male reproductive success
Source: R Soc Open Sci. 2018 Jan 17;5(1):170665. doi: 10.1098/rsos.170665 (PMC5792871; doi:10.1098/rsos.170665)
Supplement: Supplemental Report S1: Breeding behavior of male Steller sea lions in southeastern Alaska, 2000 – 2015 [file rsos170665supp1.docx]

*Supplemental material for: Hastings KK, Jemison LA, and Pendleton GW. Survival of adult Steller sea lions in Alaska: senescence, annual variation and covariation with male reproductive success. Royal Society Open Science 4:170665.*

**Supplemental Report S1: Breeding behavior of male Steller sea lions in southeastern Alaska, 2000–2015.**

Here, we present information on age-specific breeding behavior of adult male Steller sea lions (*Eumetopias jubatus*, SSL) in southeastern Alaska (SEAK) using longitudinal data, because only a single cross-sectional study on this topic has been published [1]. We also questioned the utility of data on male territorial behavior collected during boat-based resighting trips at rookeries other than Forrester Island complex (F) in SEAK, because surveys at other rookeries were late in the season (> 21 June in all years), very brief (1 to 4 days per rookery), and the timing of these surveys varied over years: from an average of 21 June (2000–2004), 19 July (2005–2006), and 1 July (2007–2015). Therefore, we first explored age-specific variation in territorial behavior of males using data collected at F in years in which in the entire breeding season was monitored to determine the impact late surveys would have on information concerning male territoriality.

First, we used daily resighting data pooled over years, to determine seasonal variation in reproductive behaviors: the proportion of pupping females in estrus and the frequency of copulations. The proportion of pupping females in estrus was calculated by adding 11 days, the average time between parturition and mating for SSL females [2–4], to the parturition date for branded females observed giving birth on Lowrie Island (L, 1 island within F), which was monitored daily (*n* = 46 females). The frequency of copulations by date was calculated by tallying by date the observed copulations of branded males and females at F, which was monitored at least weekly (*n* = 47 animals). We then examined the histories of individual branded males that were ever observed with a territory at F, and based on the seasonal patterns observed at L, we categorized male territories by their start time as prime-season (beginning before the date of the estimated maximum number of copulations, *dmc*), late season (beginning after *dmc*), and very late season (beginning after the time when 90% of copulations were observed). We then determined bias that would result from surveys after 1 July, the average survey date for boat-based surveys at rookeries other than F from 2007–2015.

At L, median parturition date of branded females was 6 June from 2007–2015, and the proportion of pups born over a 5-day period averaged 0.17 from 25 May until 18 June by which time 87% of pups had been born (figure S1). By 5 d later (23 June), 96% of pups had been born (figure S1). The seasonal pattern in the frequency of observed copulations and in the estimated proportion of pupping females in estrus was nearly identical, and peaked from 14–18 June, with only 2% of observed copulations occurring > 1 July (figure S1). We suspect the small initial peak in copulations very early in the season may have been due to copulations with females that did not pup in a given year (figure S1). Based on these patterns, we classified male territories by their start date as prime season if beginning < 19 June (black), late season if beginning > 18 June and < 1 July (blue), and very late if beginning > 30 June (red, figure S2).

Fifty-three branded males were seen at least once with territories containing females at F from 2000–2015 (figure S2*a–b*), and none of these males were observed changing breeding territories between islands at F or territorial at other rookeries in SEAK. All territorial males branded at F had the potential to reach 11 years of age by 2015. The best data was collected at L with near daily surveys for the entire breeding season. Observations of territorial males at L (*n* = 28 territorial males, ranging from ages 6–11) suggested little territorial behavior occurred < age 9 (0.02–0.07 of sightings), territorial behavior increased at age 9 (0.17 of sightings) and was highest at age 10 and 11 (0.33 and 0.37 of sightings, respectively, figure S2*a*). Eight males had intermittent (seen off and on with females) or ephemeral (< 9 days during prime season) territories in one year of their history (figure S2*a*, coded green). Average duration of territories was 35 (range 19–50), 9 and 4 days for prime, late and very late-season territories respectively (figure S2*a*). All but 3 males that were ever observed with territories (89%) had late or very late season territories (or intermittent territorial behavior, figure S2*a*) the first 1–2 years before they were observed with prime-season territories. The average start date of boat surveys at rookeries other than F in SEAK from 2007–2015 was 1 July (range 27 June – 7 July among rookeries*years). If L surveys had begun 1 July, 40% and 32% of males with prime- and late-season territories, respectively, would have already vacated their territory, and only 9% of males with very late territories would have left their territory by that date. Therefore, the timing of boat-based surveys other than at F, was too late to adequately assess the status of males during the breeding season at those rookeries.

The pattern for other islands at F was similar to that for L, with the first territorial sightings usually late season territories, of shorter duration, or intermittent compared to territories held in future years (figure S2*b*). Less temporal detail was provided for other F islands due to weekly rather than daily surveys, but these areas contained more of the oldest males from the 1994 and 1995 cohorts. Only 4 males from the 1994 and 1995 cohorts had brief territories on L (males #14–17, figure S2*a*). Ten males from these cohorts had territories on other F islands (figure S2*b*), and the last age they were seen territorial ranged to 17–18 years (figure S2*b*), also demonstrating that males can hold territories for 7–9 consecutive years. Once males hold prime-territories, nearly all continue to hold those territories in consecutive years until they are never seen again (figure S2*a–b*).

Of the 137 territorial sightings (male*year) of males at rookeries other than F, age-specific proportions were similar to L with few at ages 7 (1% of sightings) and 8 (9%), and a significant increase at age 9 (23%) and 10 (27%). For these males that were seen > 1 time per year at the rookery (*n* = 127), 35% were seen only intermittently territorial in a given year suggesting males were losing or leaving territories at the time of surveys. Several males were observed non-territorial at Graves Rocks rookery or at Inian Islands haul-out in July after holding consistent territories at F or the White Sisters rookery. Therefore daily monitoring of the entire breeding season is needed to assess territorial status of males.

One cross-sectional study of SSL found 90% of 185 territorial bulls at 4 rookeries in the Gulf of Alaska in 1959 were aged 9–13 (via canine teeth rings) [1]. Similarly, at L in SEAK, onset of territorial behavior was observed at ages 9–11, especially as late-season territories, and most males with prime-season territories were aged 11–14 (figure S2). The strategy of male SSL to hold late-season territories before prime-season territories has been noted previously [3,5]. Oldest ages for territorial males were 17–18, and, as in [3] and [5], males held territories up to 7–9 consecutive years (figure S2*b*). This pattern is similar to other polygynous species (northern elephant seals *Mirounga angustirostris* and New Zealand fur seals *Arctocephalus forsteri*), in which first ages observed territorial or copulating ranged age 8–10 with most breeding success or territorial tenure at ages 12+ [6–8]. We used this information to define prime, late and very late season territories for male SSL at F. These results also demonstrate that the timing of boat-based surveys other than at F was too late to adequately assess the status of males during the breeding season at those rookeries, and that daily monitoring of the entire breeding season is needed to accurately assess territorial status.

References

| 1. | Thorsteinson FV, Lensink CJ. 1962 Biological observations of Steller sea lions taken during an experimental harvest. *J. Wildlife Manage.* **26**, 353 – 359. |
| --- | --- |
| 2. | Gentry RL. 1970 Social behavior of the Steller sea lion. PhD thesis, University of California, Santa Cruz. |
| 3. | Parker P, Maniscalco JM. 2014 A long-term study reveals multiple reproductive behavior strategies among territorial adult male Steller sea lions (*Eumetopias jubatus*). Can. J. Zool. **92**, 405 – 415. |
| 4. | Sandegren FE. 1970 Breeding and maternal behavior of the Steller sea lion (*Eumetopias jubatus*) in Alaska. MS thesis, University of Alaska, Fairbanks. |
| 5. | Gisiner RC. 1985. Male territorial and reproductive behavior in the Steller sea lion (*Eumetopias jubatus*). PhD thesis, University of California, Santa Cruz. |
| 6. | Le Boeuf BJ, Reiter J. 1988 Lifetime reproductive success in northern elephant seals. In Reproductive success (ed TH Clutton-Brock), pp. 344 – 362. Chicago: University of Chicago Press. |
| 7. | McKenzie J, Page B, Shaughnessy PD, Hindell MA. 2007 Age and reproductive maturity of New Zealand fur seals (*Arctocephalus forsteri*) in southern Australia. J. Mammal. **88**, 639 – 648. |
| 8. | Clinton WL, Le Boeuf BJ. 1993 Sexual selection’s effects on male life history and the pattern of male mortality. Ecology **74**, 1884 – 1892. |

**Figure S1.** Timing of births, estrus and copulations for Steller sea lions during the breeding season (May–July) at Forrester Island complex, southeastern Alaska. Shown are proportions of branded females observed giving birth at Lowrie Island by date, and proportions of branded animals (males and females) observed copulating at Forrester Island complex by date (e.g. 0524 = 24 May). Proportion females in estrus by date was calculated by adding 11 days to the parturition date of females. Dashed line indicates the best visually-determined polynomial fit to the copulation data.

**Figure S2a–b.** Age-specific territorial behavior of male Steller sea lions at Lowrie Island (L, *a*) and other islands at Forrester Island complex (F, *b*), southeastern Alaska. Duration and timing of male territories per age are shown (by the position and length of line and color). Color codes: prime-season < 6/19 (black), late season >6/18 and < 7/1 (blue), very late season > 6/30 (red) and intermittent/ephemeral (green). Orange shade are poor survey years (2000–2004, 2006) and grey shade are ages not yet reached by those males (by 2015); stars indicate males seen with territory at some point during a poor survey year.


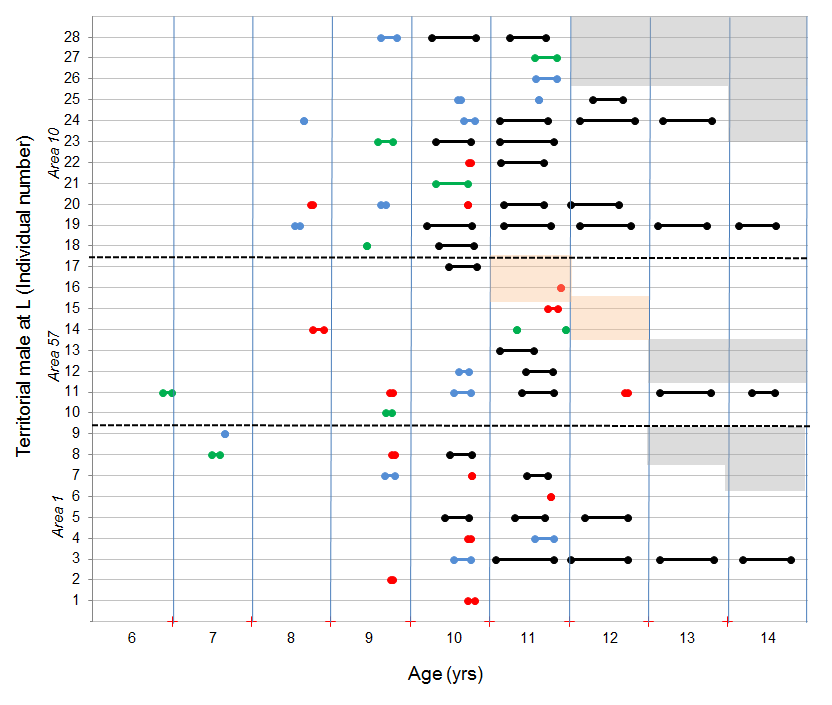


figure S2a


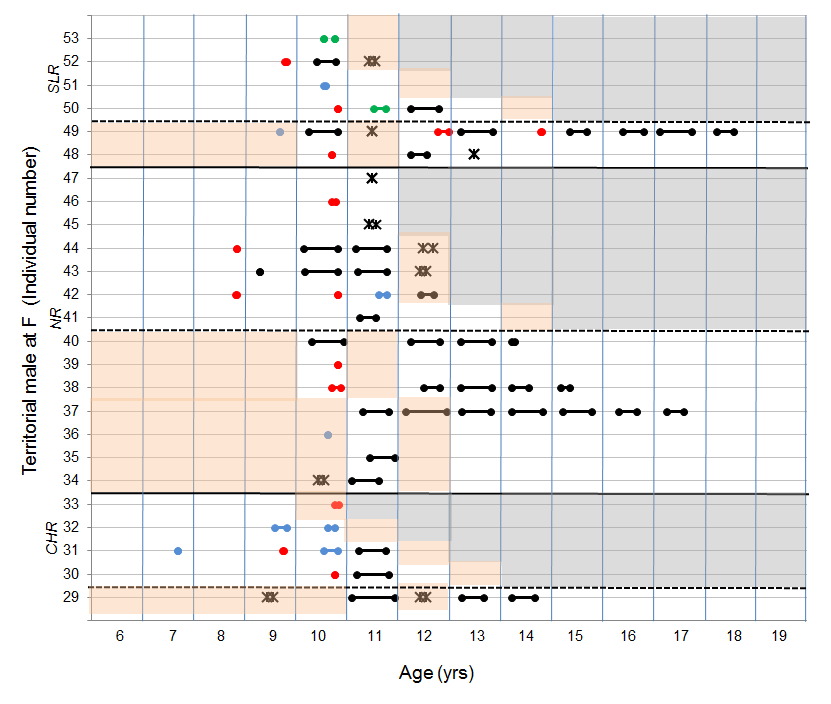


figure S2b
