# Supplementary material for: Survival of adult Steller sea lions in Alaska: senescence, annual variation and covariation with male reproductive success
Source: R Soc Open Sci. 2018 Jan 17;5(1):170665. doi: 10.1098/rsos.170665 (PMC5792871; doi:10.1098/rsos.170665)
Supplement: Supplemental Table S2. Model selection results for estimating age-specific survival of Steller sea lions in southeastern Alaska to 21 yrs of age (A), with assessment of statistical evidence of senescence (B) and annual variation (C) in adult survival, using the Cormack-Jolly-Seber model. [file rsos170665supp2.docx]

*Supplemental material for: Hastings KK, Jemison LA, and Pendleton GW. Survival of adult Steller sea lions in Alaska: senescence, annual variation and covariation with male reproductive success. Royal Society Open Science 4:170665.*

**Supplemental Table S2. Model selection results for estimating age-specific survival of Steller sea lions in southeastern Alaska to 21 yrs of age (A), with assessment of statistical evidence of senescence (B) and annual variation (C) in adult survival, using the Cormack-Jolly-Seber model.** The top ranking models are shown in (A); all models fit are shown in (B) and (C). *S* = probability of survival, *p* = probability of resighting, nr [adults] = natal rookery effect for adults only, nPar = number of parameters in the model, QAICc = Akaike's Information Criterion corrected for small sample size and overdispersion, QAICc Weight = weight of the model in relation to other models based on QAICc. In (A), 3 *S* models were fit differing only in treatment of the structure of age*sex. These 3 *S* models were fit to the same 171 *p* models; all top *p* models contained effects *p* (age*sex + year + nr[adults]) with possible age*sex structures for *p*: females (*a*: 1–3 separate, 4–14, 15+, *b*: 1–3 separate, 4+) and males (*a*: 1–8 separate, 9–12, 13+, *b*: 1–8 separate, 9+, *c*: 1–5 separate, 6–12, 13+, *d*: 1–5 separate, 6+). Age categories in *S* model 2 were: females – ages 0, 1, 2, 3 – 14 [constant annual *S* for ages 3 –14], 15 – 16, 17 – 18, 19+ vs. males – 0, 1, 2, 3 – 7, 8 – 11, 12 – 14, 15+. In (B), *S* model 2 (ages as categories) and *p* model 32 were simplified in 2 additional models, pooling the oldest age categories. In (C), 16 additional models were fit with base *S* model 3 (ages fit using B-splines) and *p* model 32 adding annual variation in males (3+ or 9+ yrs), females (3+) or both sexes 3+ (females and males pooled) born in the north (rookeries GW) and/or south (HF) southeastern Alaska (see figure 1 for rookery locations). Estimates in figure 3 are from models in red in S2-C.

| **Model#** | **Model** | **nPar** | **QAICc** | **QAICc Weight** |  |
| --- | --- | --- | --- | --- | --- |
|  |  |  |  |  |  |
| **(A) Modeling p, age*sex structure with model *p*(age*sex + year + nr[adults])** | | | | |  |
| *S* model 1: age all separate | |  |  |  |  |
| 146 | *p* (fem-b, mal-c) | 77 | 754.56 | 0.11 |  |
| 70 | *p* (fem-b, mal-c) | 78 | 754.68 | 0.10 |  |
| 127 | *p* (fem-b, mal-b) | 79 | 754.99 | 0.09 |  |
| 51 | *p* (fem-a, mal-b) | 80 | 755.38 | 0.07 |  |
| 108 | *p* (fem-b, mal-a) | 80 | 755.42 | 0.07 |  |
| 32 | *p* (fem-a, mal-a) | 81 | 755.81 | 0.06 |  |
|  |  |  |  |  |  |
| *S* model 2: age categories | |  |  |  |  |
| 70 | *p* (fem-a, mal-c) | 55 | 722.29 | 0.11 |  |
| 51 | *p* (fem-a, mal-b) | 57 | 722.51 | 0.10 |  |
| 32 | *p* (fem-a, mal-a) | 58 | 722.88 | 0.08 |  |
| 127 | *p* (fem-b, mal-b) | 56 | 722.89 | 0.08 |  |
| 146 | *p* (fem-b, mal-c) | 54 | 722.97 | 0.08 |  |
| 108 | *p* (fem-b, mal-a) | 57 | 723.26 | 0.07 |  |
|  |  |  |  |  |  |
| *S* model 3: age modeled as B-splines | |  |  |  |  |
| 127 | *p* (fem-b, mal-b) | 53 | 720.61 | 0.10 |  |
| 108 | *p* (fem-b, mal-a) | 54 | 720.93 | 0.09 |  |
| 51 | *p* (fem-a, mal-b) | 54 | 720.93 | 0.09 |  |
| 32 | *p* (fem-a, mal-a) | 55 | 721.25 | 0.07 |  |
| 146 | *p* (fem-b, mal-c) | 51 | 721.25 | 0.07 |  |
| 70 | *p* (fem-a, mal-c) | 52 | 721.30 | 0.07 |  |
|  |  |  |  |  |  |
| **(B) Senescence in *S*, with *S* model 2: age categories and *p* model 32** | | | | |  |
| *S.*2-*p.*32 | Base | 58 | 722.88 | 0.76 |  |
| 172 | *S* (fem only - no senescence) | 55 | 725.19 | 0.23 |  |
| 173 | *S* (mal only - no senescence) | 56 | 751.01 | 0.01 |  |
|  |  |  |  |  |  |
| **(C) Annual variation in adult *S*, with *S* model 3: B-splines and *p* model 32** | | | | |  |
| 180 | *S* (mal 3+: north) | 65 | 716.85 | 0.79 |  |
| 184 | *S* (mal 9+: north) | 59 | 720.93 | 0.10 |  |
| *S.*3-*p*.32 | Base | 55 | 721.25 | 0.09 |  |
| 188 | *S* (adults 3+: north) | 65 | 723.78 | 0.02 |  |
| 176 | *S* (fem 3+: north) | 65 | 732.22 | 0.00 |  |
| 183 | *S* (mal 9+: south) | 67 | 734.22 | 0.00 |  |
| 182 | *S* (mal 9+: south and north) | 71 | 734.59 | 0.00 |  |
| 178 | *S* (mal 3+: south and north) | 80 | 735.64 | 0.00 |  |
| 185 | *S* (mal 9+) | 67 | 736.12 | 0.00 |  |
| 179 | *S* (mal 3+: south) | 70 | 736.82 | 0.00 |  |
| 187 | *S* (adults 3+: south) | 70 | 739.21 | 0.00 |  |
| 175 | *S* (fem 3+: south) | 70 | 739.36 | 0.00 |  |
| 181 | *S* (mal 3+) | 70 | 741.65 | 0.00 |  |
| 177 | *S* (fem 3+) | 70 | 743.40 | 0.00 |  |
| 189 | *S* (adults 3+) | 70 | 745.26 | 0.00 |  |
| 186 | *S* (adults 3+: south and north) | 80 | 747.06 | 0.00 |  |
| 174 | *S* (fem 3+: south and north) | 80 | 752.28 | 0.00 |  |
