# Supplementary material for: Survival of adult Steller sea lions in Alaska: senescence, annual variation and covariation with male reproductive success
Source: R Soc Open Sci. 2018 Jan 17;5(1):170665. doi: 10.1098/rsos.170665 (PMC5792871; doi:10.1098/rsos.170665)
Supplement: Supplemental Table S3. Age- and sex-specific survival probabilities (S ^) of Steller sea lions in southeastern Alaska by natal rookery. [file rsos170665supp3.docx]

*Supplemental material for: Hastings KK, Jemison LA, and Pendleton GW. Survival of adult Steller sea lions in Alaska: senescence, annual variation and covariation with male reproductive success. Royal Society Open Science 4:170665.*

**Supplemental Table S3. Age- and sex-specific survival probabilities (**$\hat{\boldsymbol{S}}$**) of Steller sea lions in southeastern Alaska by natal rookery.** Natal rookeries were: F = Forrester Island complex, H = Hazy Islands, W = White Sisters, G = Graves Rocks (see figure 1). $\hat{S}$ are from S model 3, model 32, in Supplemental Table S2-A. For ages 0 and 1, estimates shown are for the 2004 cohorts for F and W, the 2003 cohort for H and the 2002 cohort for G. se = standard error of $\hat{S}$, lcl/ucl = lower and upper confidence limits for $\hat{S}$.

|  | F | | | | H | | | | W | | | | G | | | |
| --- | --- | --- | --- | --- | --- | --- | --- | --- | --- | --- | --- | --- | --- | --- | --- | --- |
| age | $\hat{S}$ | se | lcl | ucl | $\hat{S}$ | se | lcl | ucl | $\hat{S}$ | se | lcl | ucl | $\hat{S}$ | se | lcl | ucl |
| females |  |  |  |  |  |  |  |  |  |  |  |  |  |  |  |  |
| 0 | 0.561 | 0.031 | 0.500 | 0.620 | 0.573 | 0.033 | 0.507 | 0.637 | 0.663 | 0.032 | 0.597 | 0.723 | 0.720 | 0.039 | 0.639 | 0.789 |
| 1 | 0.763 | 0.017 | 0.729 | 0.793 | 0.771 | 0.024 | 0.722 | 0.814 | 0.832 | 0.016 | 0.798 | 0.861 | 0.866 | 0.022 | 0.817 | 0.904 |
| 2 | 0.864 | 0.012 | 0.839 | 0.886 | 0.874 | 0.013 | 0.846 | 0.897 | 0.907 | 0.010 | 0.885 | 0.926 | 0.934 | 0.012 | 0.907 | 0.953 |
| 3 | 0.911 | 0.009 | 0.892 | 0.928 | 0.918 | 0.009 | 0.897 | 0.935 | 0.941 | 0.007 | 0.924 | 0.954 | 0.958 | 0.008 | 0.940 | 0.971 |
| 4 | 0.933 | 0.007 | 0.918 | 0.946 | 0.938 | 0.007 | 0.922 | 0.951 | 0.955 | 0.006 | 0.943 | 0.965 | 0.968 | 0.006 | 0.955 | 0.978 |
| 5 | 0.942 | 0.006 | 0.930 | 0.953 | 0.947 | 0.006 | 0.934 | 0.957 | 0.962 | 0.005 | 0.951 | 0.970 | 0.973 | 0.005 | 0.961 | 0.981 |
| 6 | 0.945 | 0.005 | 0.933 | 0.954 | 0.949 | 0.006 | 0.937 | 0.959 | 0.963 | 0.005 | 0.953 | 0.971 | 0.974 | 0.005 | 0.963 | 0.982 |
| 7 | 0.943 | 0.006 | 0.930 | 0.953 | 0.947 | 0.006 | 0.934 | 0.958 | 0.962 | 0.005 | 0.951 | 0.971 | 0.973 | 0.005 | 0.961 | 0.982 |
| 8 | 0.938 | 0.007 | 0.923 | 0.950 | 0.943 | 0.007 | 0.927 | 0.955 | 0.959 | 0.006 | 0.946 | 0.969 | 0.971 | 0.006 | 0.957 | 0.980 |
| 9 | 0.931 | 0.008 | 0.913 | 0.946 | 0.936 | 0.009 | 0.917 | 0.951 | 0.954 | 0.007 | 0.939 | 0.966 | 0.968 | 0.006 | 0.952 | 0.978 |
| 10 | 0.925 | 0.009 | 0.905 | 0.940 | 0.930 | 0.010 | 0.909 | 0.947 | 0.950 | 0.008 | 0.933 | 0.963 | 0.964 | 0.007 | 0.947 | 0.976 |
| 11 | 0.920 | 0.010 | 0.899 | 0.937 | 0.926 | 0.010 | 0.903 | 0.944 | 0.946 | 0.008 | 0.928 | 0.960 | 0.962 | 0.008 | 0.944 | 0.975 |
| 12 | 0.916 | 0.011 | 0.893 | 0.935 | 0.923 | 0.012 | 0.897 | 0.943 | 0.944 | 0.009 | 0.924 | 0.959 | 0.960 | 0.008 | 0.940 | 0.974 |
| 13 | 0.914 | 0.013 | 0.884 | 0.936 | 0.920 | 0.014 | 0.889 | 0.943 |  |  |  |  |  |  |  |  |
| 14 | 0.910 | 0.016 | 0.872 | 0.937 |  |  |  |  |  |  |  |  |  |  |  |  |
| 15 | 0.903 | 0.019 | 0.858 | 0.935 |  |  |  |  |  |  |  |  |  |  |  |  |
| 16 | 0.892 | 0.022 | 0.842 | 0.928 |  |  |  |  |  |  |  |  |  |  |  |  |
| 17 | 0.874 | 0.025 | 0.817 | 0.915 |  |  |  |  |  |  |  |  |  |  |  |  |
| 18 | 0.842 | 0.034 | 0.762 | 0.898 |  |  |  |  |  |  |  |  |  |  |  |  |
| 19 | 0.788 | 0.065 | 0.634 | 0.889 |  |  |  |  |  |  |  |  |  |  |  |  |
| 20 | 0.699 | 0.134 | 0.400 | 0.890 |  |  |  |  |  |  |  |  |  |  |  |  |
|  |  |  |  |  |  |  |  |  |  |  |  |  |  |  |  |  |
| males |  |  |  |  |  |  |  |  |  |  |  |  |  |  |  |  |
| 0 | 0.511 | 0.031 | 0.450 | 0.571 | 0.523 | 0.033 | 0.458 | 0.587 | 0.617 | 0.034 | 0.549 | 0.680 | 0.678 | 0.042 | 0.592 | 0.753 |
| 1 | 0.692 | 0.020 | 0.652 | 0.729 | 0.702 | 0.028 | 0.645 | 0.754 | 0.776 | 0.020 | 0.735 | 0.812 | 0.819 | 0.028 | 0.757 | 0.868 |
| 2 | 0.797 | 0.017 | 0.762 | 0.828 | 0.810 | 0.018 | 0.772 | 0.843 | 0.858 | 0.015 | 0.826 | 0.885 | 0.896 | 0.017 | 0.857 | 0.926 |
| 3 | 0.850 | 0.013 | 0.823 | 0.874 | 0.861 | 0.014 | 0.832 | 0.886 | 0.897 | 0.011 | 0.873 | 0.917 | 0.926 | 0.013 | 0.897 | 0.947 |
| 4 | 0.875 | 0.011 | 0.852 | 0.894 | 0.884 | 0.011 | 0.860 | 0.904 | 0.915 | 0.009 | 0.895 | 0.931 | 0.939 | 0.010 | 0.915 | 0.956 |
| 5 | 0.881 | 0.011 | 0.858 | 0.901 | 0.890 | 0.011 | 0.866 | 0.910 | 0.920 | 0.009 | 0.900 | 0.936 | 0.942 | 0.010 | 0.920 | 0.959 |
| 6 | 0.875 | 0.013 | 0.848 | 0.898 | 0.884 | 0.013 | 0.856 | 0.907 | 0.915 | 0.011 | 0.892 | 0.934 | 0.939 | 0.011 | 0.914 | 0.957 |
| 7 | 0.857 | 0.015 | 0.826 | 0.883 | 0.867 | 0.015 | 0.834 | 0.894 | 0.902 | 0.012 | 0.875 | 0.924 | 0.930 | 0.013 | 0.901 | 0.951 |
| 8 | 0.826 | 0.015 | 0.793 | 0.854 | 0.838 | 0.017 | 0.802 | 0.868 | 0.879 | 0.014 | 0.850 | 0.904 | 0.913 | 0.015 | 0.879 | 0.938 |
| 9 | 0.783 | 0.019 | 0.743 | 0.818 | 0.797 | 0.021 | 0.754 | 0.834 | 0.847 | 0.018 | 0.810 | 0.879 | 0.888 | 0.019 | 0.846 | 0.920 |
| 10 | 0.733 | 0.027 | 0.677 | 0.782 | 0.749 | 0.028 | 0.690 | 0.800 | 0.808 | 0.024 | 0.756 | 0.852 | 0.858 | 0.025 | 0.801 | 0.901 |
| 11 | 0.683 | 0.034 | 0.613 | 0.746 | 0.701 | 0.036 | 0.627 | 0.766 | 0.768 | 0.032 | 0.701 | 0.824 | 0.826 | 0.032 | 0.754 | 0.881 |
| 12 | 0.645 | 0.042 | 0.558 | 0.724 | 0.664 | 0.044 | 0.574 | 0.744 | 0.737 | 0.040 | 0.652 | 0.807 | 0.801 | 0.039 | 0.712 | 0.867 |
| 13 | 0.632 | 0.071 | 0.486 | 0.757 | 0.651 | 0.071 | 0.503 | 0.775 |  |  |  |  |  |  |  |  |
| 14 | 0.652 | 0.130 | 0.378 | 0.852 |  |  |  |  |  |  |  |  |  |  |  |  |
| 15+ | 0.570 | 0.148 | 0.289 | 0.812 |  |  |  |  |  |  |  |  |  |  |  |  |
