# Supplementary material for: Survival of adult Steller sea lions in Alaska: senescence, annual variation and covariation with male reproductive success
Source: R Soc Open Sci. 2018 Jan 17;5(1):170665. doi: 10.1098/rsos.170665 (PMC5792871; doi:10.1098/rsos.170665)
Supplement: Supplemental Table S4. Model selection results for survival differences between territorial and non-territorial Steller sea lion males in southeastern Alaska (2007–15) using the multi-state model. [file rsos170665supp4.docx]

*Supplemental material for: Hastings KK, Jemison LA, and Pendleton GW. Survival of adult Steller sea lions in Alaska: senescence, annual variation and covariation with male reproductive success. Royal Society Open Science 4:170665.*

**Supplemental Table S4. Model selection results for survival differences between territorial and non-territorial Steller sea lion males in southeastern Alaska (2007–2015) using the multi-state model.** Best data were provided by males born at Forrester Islands (F, T(F) = territorial at F, N = non-territorial at F) which were monitored the entire breeding season. Territorial status of males at haul-outs and other rookeries (T* = territorial at other rookery, H* = only seen non-territorial at other rookery or haul-out) was less certain due to surveys being conducted only at the very end of or just after the breeding season (> 1 July), and therefore these uncertain categories were considered separately from F data. The same 7 models were fit to 3 datasets in which for F data, the type of territory was considered in categorizing males as T(F) or N: A = prime-season territory (beginning <19 June), B = late season territory (beginning >18–30 June), and C = very late season (>30 June; see Supplemental Report S1). Model = base survival model fit to male data only: *S* (age + nr) with age fit using B-splines (S model 3 in Supplemental Table 2A) with addition of stratum (territorial status) effects. See text for explanation of models used for ψ (probability of transitioning between states). Resighting rate model was *p* (mal-d + year + nr[adults] + stratum) where mal-d was an age structure for males explained in Supplemental Table S2 caption. nPar = number of parameters in the model, AICc = Akaike's Information Criterion corrected for small sample size, AICc Weight = weight of the model in relation to other models based on AICc.

| **Model#** | **Model** | **nPar** | **AICc** | **AICc Weight** |
| --- | --- | --- | --- | --- |
| (Dataset 1) T(F) = A+B+C, N= N; T(F) includes all territorial behavior† | | | |  |
| 5 | H*=T*=N + T(F) | 77 | 306.02 | 0.41 |
| 3 | H*=N + T* + T(F) | 78 | 307.51 | 0.19 |
| 6 | H*=T* + N + T(F) | 78 | 307.91 | 0.16 |
| 7 | all the same: H*=T*=N=T(F) | 76 | 309.07 | 0.09 |
| 1 | all different: H* + T* + N + T(F) | 79 | 309.31 | 0.08 |
| 4 | H*=N + T*=T(F) | 77 | 310.85 | 0.04 |
| 2 | H* + T* + N=T(F) | 78 | 311.16 | 0.03 |
|  |  |  |  |  |
| (Dataset 2) T(F) = A+B, N = N+C, T(F) excludes males with very late season territories‡ | | | | |
| 5 | H*=T*=N + T(F) | 79 | 305.83 | 0.30 |
| 7 | all the same: H*=T*=N=T(F) | 78 | 306.44 | 0.22 |
| 3 | H*=N + T* + T(F) | 80 | 307.08 | 0.16 |
| 6 | H*=T* + N + T(F) | 80 | 307.89 | 0.11 |
| 2 | H* + T* + N=T(F) | 80 | 308.46 | 0.08 |
| 4 | H*=N + T*=T(F) | 79 | 308.53 | 0.08 |
| 1 | all different: H* + T* + N + T(F) | 81 | 309.16 | 0.05 |
|  |  |  |  |  |
| (Dataset 3) T(F) = A, N = N+B+C, T(F) includes only males with prime season territoriesⁿ | | | | |
| 7 | all the same: H*=T*=N=T(F) | 76 | 286.05 | 0.33 |
| 5 | H*=T*=N + T(F) | 77 | 287.15 | 0.19 |
| 4 | H*=N + T*=T(F) | 77 | 287.90 | 0.13 |
| 2 | H* + T* + N=T(F) | 78 | 288.07 | 0.12 |
| 3 | H*=N + T* + T(F) | 78 | 288.14 | 0.11 |
| 6 | H*=T* + N + T(F) | 78 | 288.82 | 0.08 |
| 1 | all different: H* + T* + N + T(F) | 79 | 289.98 | 0.04 |

† Best ψ: T*/T(F) = no age, H*/N = age3, model weight = 0.92, next best model weight = 0.06

‡ Best ψ: T(F) = age2, T* = no age, H*/N = age3, model weight = 0.61, next best model weight = 0.22

ⁿ Best ψ: T*/T(F) = no age, H*/N = age3, model weight = 0.92, next best model weight = 0.06
