# Supplementary material for: Survival of adult Steller sea lions in Alaska: senescence, annual variation and covariation with male reproductive success
Source: R Soc Open Sci. 2018 Jan 17;5(1):170665. doi: 10.1098/rsos.170665 (PMC5792871; doi:10.1098/rsos.170665)
Supplement: Supplemental Table S5. Estimates of the probability of Steller sea lion males transitioning between territorial states (ψ) in southeastern Alaska, 2007–2015 by age and natal rookery. [file rsos170665supp5.docx]

*Supplemental material for: Hastings KK, Jemison LA, and Pendleton GW. Survival of adult Steller sea lions in Alaska: senescence, annual variation and covariation with male reproductive success. Royal Society Open Science 4:170665.*

**Supplemental Table S5. Estimates of the probability of Steller sea lion males transitioning between territorial states (ψ) in southeastern Alaska, 2007–2015 by age and natal rookery.** Estimates are from model 5 in Supplemental Table S4 (see Supplemental Table S4 footnotes and caption for explanation of datasets and codes: H*, T*, N, T[F]). Transitioning between T* and T(F) was fixed to 0 because no males were observed with territories at more than one rookery. Age effects are indicated as Young/Midage/Old for age3 models (age3 = 3 categories: 5–7, 8–9, 10+ yrs); and by Younger/Old for age2 models (age2 = 2 categories: 5–9, 10+). Transitions with a single value are from "no age" models. When estimates were the same for all datasets 1–3, single estimates are shown for dataset="all".

|  |  | To |  |  |  |
| --- | --- | --- | --- | --- | --- |
| From | *Dataset* | H* | T* | T(F) | N |
| **F males** |  |  |  |  |  |
| H* | *all* | 0.74 - 0.84 - 0.92 | 0.01 - 0.09 - 0.08 | 0 | 0.25 - 0.07 - 0.00 |
| T* | *all* | 0.61 | 0.39 | *Fixed=0* | 0 |
|  |  |  |  |  |  |
|  | *1* | 0 | *Fixed=0* | 0.88 | 0.12 |
| T(F) | *2* | 0 | *Fixed=0* | 0.24, 0.96 | 0.67, 0.04 |
|  | *3* | 0 | *Fixed=0* | 0.95 | 0.05 |
|  |  |  |  |  |  |
|  | *1* | 0.13 - 0.00 - 0.00 | 0 | 0.03 - 0.43 - 1.00 | 0.84 - 0.57 - 0.00 |
| N | *2* | 0.13 - 0.00 - 0.00 | 0 | 0.03 - 0.25 - 0.58 | 0.84 - 0.75 - 0.42 |
|  | *3* | 0.13 - 0.00 - 0.00 | 0 | 0.00 - 0.12 - 0.61 | 0.87 - 0.88 - 0.39 |
|  |  |  |  |  |  |
| **H males** |  |  |  |  |  |
| H* | *all* | 0.98 - 0.79 - 0.79 | 0.02 - 0.21 - 0.21 |  |  |
| T* | *all* | 0.36 | 0.64 |  |  |
|  |  |  |  |  |  |
| **WG males** | |  |  |  |  |
| H* | *all* | 0.96 - 0.76 - 0.82 | 0.04 - 0.24 - 0.18 |  |  |
| T* | *all* | 0.30 | 0.70 |  |  |
